# Sleep Deprivation Aggravates Cognitive Impairment by the Alteration of Hippocampal Neuronal Activity and the Density of Dendritic Spine in Isoflurane-Exposed Mice

**DOI:** 10.3389/fnbeh.2020.589176

**Published:** 2020-11-23

**Authors:** Kai Zhang, Naqi Lian, Ran Ding, Cunle Guo, Xi Dong, Yuanyuan Li, Sheng Wei, Qingyan Jiao, Yonghao Yu, Hui Shen

**Affiliations:** ^1^Department of Anesthesia, Tianjin Medical University General Hospital, Tianjin, China; ^2^Tianjin Institute of Anesthesiology, Tianjin, China; ^3^Chinese Institute for Brain Research, Beijing (CIBR), Beijing, China; ^4^Laboratory of Neurobiology, School of Biomedical Engineering, Tianjin Medical University, Tianjin, China; ^5^Experimental Center, Shandong University of Traditional Chinese Medicine, Jinan, China; ^6^Institute of Neurology, Tianjin Medical University General Hospital, Tianjin, China

**Keywords:** isoflurane, sleep deprivation, short-term memory, long-term memory, Ca^2+^ signals, dendritic spine

## Abstract

Isoflurane contributes to cognitive deficits when used as a general anesthetic, and so does sleep deprivation (SD). Patients usually suffer from insomnia before an operation due to anxiety, fear, and other factors. It remains unclear whether preoperative SD exacerbates cognitive impairment induced by isoflurane. In this study, we observed the effects of pretreated 24-h SD in adult isoflurane-exposed mice on the cognitive behaviors, the Ca^2+^ signals of dorsal hippocampal CA1 (dCA1) neurons *in vivo* with fiber photometry, and the density of dendritic spines in hippocampal neurons. Our results showed that in cognitive behavior tasks, short-term memory damages were more severe with SD followed by isoflurane exposure than that with SD or isoflurane exposure separately, and interestingly, severe long-term memory deficits were induced only by SD followed by isoflurane exposure. Only the treatment of SD followed by isoflurane exposure could reversibly decrease the amplitude of Ca^2+^ signals when mice were freely moving and increase the duration of Ca^2+^ signals during the long-term memory behavior test. The density of dendritic spines with both SD and isoflurane exposure was lower than that with SD alone. This study suggests that SD should be avoided preoperatively in patients undergoing elective surgery under isoflurane anesthesia.

## Introduction

Isoflurane is one of the commonly used anesthetics in the operating room, but there is a lot of evidence that it could lead to transient cognitive impairment (Cao et al., 2019; Schaefer et al., 2019; Song et al., 2019; Zhang and Yuan, 2019), and some studies even believe that its cognitive impairment can persist for a long time (Culley et al., 2004; Landin et al., 2019). Although most studies have been limited to developmental and old age, research that the cognitive function of adult mice is susceptible to isoflurane intervention has also been reported (Lin and Zuo, 2011; Pearce et al., 2012; Zhang et al., 2018). Sleep plays an extremely important role in promoting physical and mental health. Clinical trials have shown that sleep significantly improves memory consolidation compared to the same length of wakefulness and lack of sleep will cause lack of concentration and work efficiency (Stickgold, 2005; Payne et al., 2012). Animal studies reveal that with prolonged sleep deprivation (SD), different cognitive functions, including learning abilities, and spatial memory are impaired to a greater degree (Guan et al., 2004; Wang et al., 2009). Unfortunately, SD has become an increasingly common phenomenon in people's daily lives, especially in patients with anxiety, fear, and other negative emotions due to clinical surgery, which often seriously affect the quality and duration of sleep. However, there has been no reported interaction between isoflurane exposure and SD on damage of cognitive function. In the current study, we aimed to explore whether 24-h SD would impact Post-anesthesia cognition deficits.

Dorsal hippocampus especially contributes to spatial information and other cognitive functions (Strange et al., 2014). Both isoflurane exposure and SD have been well-documented to cause pathological changes in the hippocampus at cellular and molecular levels, including changing the expression of inflammatory proteins, signaling molecules, and oxidative stress factors (Yuan et al., 2019; Li et al., 2020; Wen et al., 2020). However, these studies are much limited to hippocampal neurons *in vitro*. Population neuronal activity is considered to contribute to various brain functions, including learning and memory (Rolls et al., 2011). It is generally accepted that recording Ca^2+^ signals represents an effective method for exploring the properties of such integrated cell activity *in vivo* in neurons where Ca^2+^ transients allow the representation of spiking activity (Mao et al., 2001). Here we employed a technology termed fiber photometry, which allows to monitor Ca^2+^ signals for deep tissue in freely moving animals. In fact, this technique has been used to study cortical neuronal activity in isoflurane-anesthetized and freely moving states in mice (Zhang et al., 2017). In the present study, we observed the effects of isoflurane and SD on dorsal hippocampus CA1 (dCA1) neuronal Ca^2+^ signals in mice during free movement and the relationship between the Ca^2+^ signals and cognitive behavioral tasks.

Dendritic spines are Post-synaptic structures at a majority of excitatory synapses in mammalian brain (Rochefort and Konnerth, 2012). The number and size of dendritic spines are closely related to cognitive function in different neurological diseases (Blanpied and Ehlers, 2004). The changed number of dendritic spines induced by anesthetic or SD on the synaptic plasticity is becoming an arresting area. Previous studies have revealed that isoflurane could reduce hippocampal dendritic spines in both adult and developmental mice (Platholi et al., 2014; Schaefer et al., 2019). However, the effects of SD on hippocampal dendritic spines have not been finalized. Accumulating evidence supports that SD leads to upregulation of dendritic spines (de Vivo et al., 2017) and driven mainly by greater numbers of immature spines (Gisabella et al., 2020), while other studies summarized a loss of spines in the CA1 region following 5-h SD (Havekes et al., 2016). Therefore, on one hand, we wanted to know the actual effect of SD on hippocampal dendritic spines, and on the other hand, we aimed to observe the effect of SD on the change of spines caused by isoflurane.

## Materials and Methods

### Animals

Male C57BL/6J mice (8–10 weeks, 20~25 g) were used in this study. Animals were randomly assigned into four groups: control group (*n* = 14); isoflurane group (*n* = 14); SD group (*n* = 14); SD + isoflurane group (*n* = 14). Three or four mice were fed in each cage, except that mice implanted with optical fibers were single-caged. The animals were free to access water and food (except for those used for radial arm maze task) and were maintained under the condition of a 12-h light–dark cycle (lights on at 7:00 a.m.). The room temperature was 23°C ± 2°C and the humidity was 50% ± 10%. Behavioral tests were conducted during the light period. All the experimental processes were performed in compliance with National Institutes of Health guidelines and overseen by the Animal Care and Use Committee of Tianjin Medical University.

### Isoflurane Exposure

Mice were placed in a homemade anesthetic chamber and primed with 100% oxygen (O_2_) containing 2.5% isoflurane for 5 min. Then, the concentration of isoflurane was maintained with 1.5% mixed with 2 L/min pure O_2_ for 4 h. The concentrations of isoflurane and end-expiratory carbon dioxide were continuously monitored by a gas monitor. Mice received pure O_2_ for 5 min at the end of isoflurane inhalation and then were returned to their original cages. Mice in control and SD groups breathed in free air.

### Sleep Deprivation

Mice were subjected to 24-h SD using a multiple platform water environment SD device (ZS Dichuang, China). There were 10 platforms with the diameter and height of each being 3 and 5 cm, respectively, Clean water was poured 1 cm below the platforms when mice were put on the platforms. The animals without SD treatment were applied with the same procedure except that the platforms were 8 cm in diameter. A heating device was used to keep water temperature at 25°C.

### *In vivo* Optical Fiber Photometry for Ca^2+^ Recordings

#### Surgery and Virus Injection

Mice were anesthetized with isoflurane–oxygen mixture (1.2% vol. isoflurane/vol. O_2_). After the skin was cut off, a diminutive craniotomy (0.5 mm × 0.5 mm) was made unilaterally above the dorsal hippocampus at −1.8 mm anteroposterior (AP) and 1.5 mm mediolateral (ML) from the bregma with a dental drill. A glass micropipette with a tip diameter of about 15 μm filled with ~150 nl of pAAV-Syn-GCaMP6f virus solution was implanted into the target tissue at a depth of 1.25 mm dorsoventrally (DV, from the endocranium surface). Following the injection, the micropipette remained stationary for 10 min to ensure the virus spread sufficiently before being pulled out slowly. Thereafter, a 200-μm-diameter optical fiber threaded through a ceramic ferrule with the fiber tip extended 1.8 mm out of the ferrule (Shanghai Fiblaser, China) was inserted through the same craniotomy and advanced slowly to a little above the virus injection point. Finally, the cannula was fixed to the skull with dental cement. When the cement dried, each mouse was placed to its cage and raised alone; mice received intraperitoneal injection of analgesics for 3 days to aid recovery.

#### Optical Fiber-Based Ca^2+^ Recordings

A custom-built device was applied for neuronal Ca^2+^ signal measurements (model “Fiber OptoMeter v2.0,” Suzhou Institute of Biomedical Engineering and Technology). pAAV-Syn-GCaMP6f was activated by light from a solid-state laser (470 nm, Coherent). The light intensity of the tip of the fiber was ~0.22 μW/mm^2^. Ca^2+^ signals were digitized at 2,000 Hz with custom-written data acquisition software based on LabVIEW (National Instruments, Austin, TX, USA; Suzhou Institute of Biomedical Engineering and Technology). Simultaneously, behaviors of mice were captured, and the videos were recorded at 30 Hz with a spatial resolution of 1,280 × 720 pixels (Logitech C270, Switzerland). All Ca^2+^ signals and behavioral videos were synchronized offline with event marks.

#### Histology and Fluorescence Imaging

When all the experiments were completed, mice that had been injected with the virus were perfused transcardially with 4% paraformaldehyde (PFA) in phosphate-buffered saline (PBS) to confirm the viral expression and the position of the fiber implantation. Brain samples were soaked in 4% PFA and stored overnight at 4°C, then they were sectioned into 70-μm slices using a vibratome (Leica, USA). Images were obtained using an inverted fluorescence microscope (BX51, Olympus).

### Behavioral Tests

#### Optical Fiber Ca^2+^ Signals Recordings in Freely Moving Mice

Mice were placed in a glass chamber and allowed to move freely. A camera was put just above the chamber and captured the activities of mice. We continuously recorded each mouse for 5 min. To minimize the interference of environment and investigator on hippocampal neuronal excitability, mice were stroked by the investigator for 5 min and placed in the chamber to adapt for 10 min each day for 3 days prior to the experiments. Additional 5-min acclimation in the chamber was permitted before recordings on the testing day, and the surrounding environment remained basically unchanged during the whole process.

#### Y Maze Test

The Y maze (ZS Dichuang, China) was used to assess short-term memory in mice (Kraeuter et al., 2019). There were three interconnected arms (120° angle between arms) with each measuring 30 cm × 6 cm × 20 cm (length × width × height). The three arms were randomly defined as the novel arm, the other arm, and the start arm. This behavioral experiment consisted of training and test phases. Three days after habituation in the experimental room, the training phases were performed 2 h after treatments. During the 10-min training phase, one of the three arms was closed off. Mice were then removed and 1 h later replaced in the Y maze (with all arms open and mice scents removed) for the 5-min test phase. This task is based on the natural tendency of rodents to explore novel environments, thus, if mice recognize the already explored arms, they should spend more time exploring the novel arm. The greater time that mice explore the novel arm represents greater memory. The whole process of this task was recorded by a camera, and Ca^2+^ signals of dCA1 were simultaneously recorded.

#### Radial Arm Maze Test

Radial arm (Dichuang, China) was applied to evaluate spatial reference memory, which depends on long-term memory (Bannerman et al., 2014). The maze contains eight arms radiated from the maze center, with each being 30 cm × 6 cm × 20 cm (length × width × height). There was a small and same food grooved trough at the end of each arm, but we only put food (a small piece of raw peanut) in one arm. Three days after habituation in the experimental room, mice were trained 5 times/day (1-h interval) for about 7 consecutive days. The well-trained performance was that mice could attain food with no errors (the number of times that mice entered non-placed food arms) and with searching time <10 s. We excluded mice that had not learned and that were inactive more than 20% searching time during the task. Then, mice were randomly divided into the four groups. Two hours after their own treatments, they were put back into the radial maze for the test phase. The movements of mice were captured by a camera, and Ca^2+^ signals of dCA1 were simultaneously recorded.

### Golgi Staining and Spines Measurement

Brains obtained from euthanized mice (four mice each group) were processed for Golgi impregnation using the FD Rapid Golgi Stain Kit (FD NeuroTechnologies, USA). Then, the tissues were coronally sectioned into a thickness of 100 μm with a vibratome. After overnight, all sections were sequentially stained according to the manufacturer's procedure and stored in darkness. The imaging of pyramidal cells from dCA1 neurons and subsequent counts were carried out by a single, blinded experimenter. The neurons were viewed with a 100 × oil objective on an upright light microscope, and five cells were imaged from each brain sample. We preferred neurons that had a clear field of vision and did not overlap with other cellular dendrites. We adjusted the contrast of the microscope image to count the dendritic spines more clearly. We analyzed the spines on both apical and basal secondary dendrites. Quantitative analysis used the ImageJ software (Open Source from National Institutes of Health, Bethesda, MD).

### Analysis and Statistics

We analyzed fiber data using the procedure similar to the previous work (Qin et al., 2018). The changes of relative fluorescence ΔF/F = (f - f_baseline_)/f_baseline_ were represented as Ca^2+^ signals, where the f_baseline_ was the basal level of fluorescence calculated during the whole recording period. A Ca^2+^ signal was accepted when its amplitude was five times the standard deviation of the control time. All data were expressed as mean ± standard error (mean ± SEM), and analysis was conducted using Prism 7 (GraphPad Software Inc., CA, USA). We used three methods throughout this study, including paired *t*-test for paired two-group analyses, RM one-way ANOVA, and Tukey test for paired multi-group analyses or one-way ANOVA and Tukey test for unpaired multi-group analyses.

## Results

### Mice in SD+Isoflurane Group Showed More Severe Short-Term Memory Impairment

**Figure 1:**
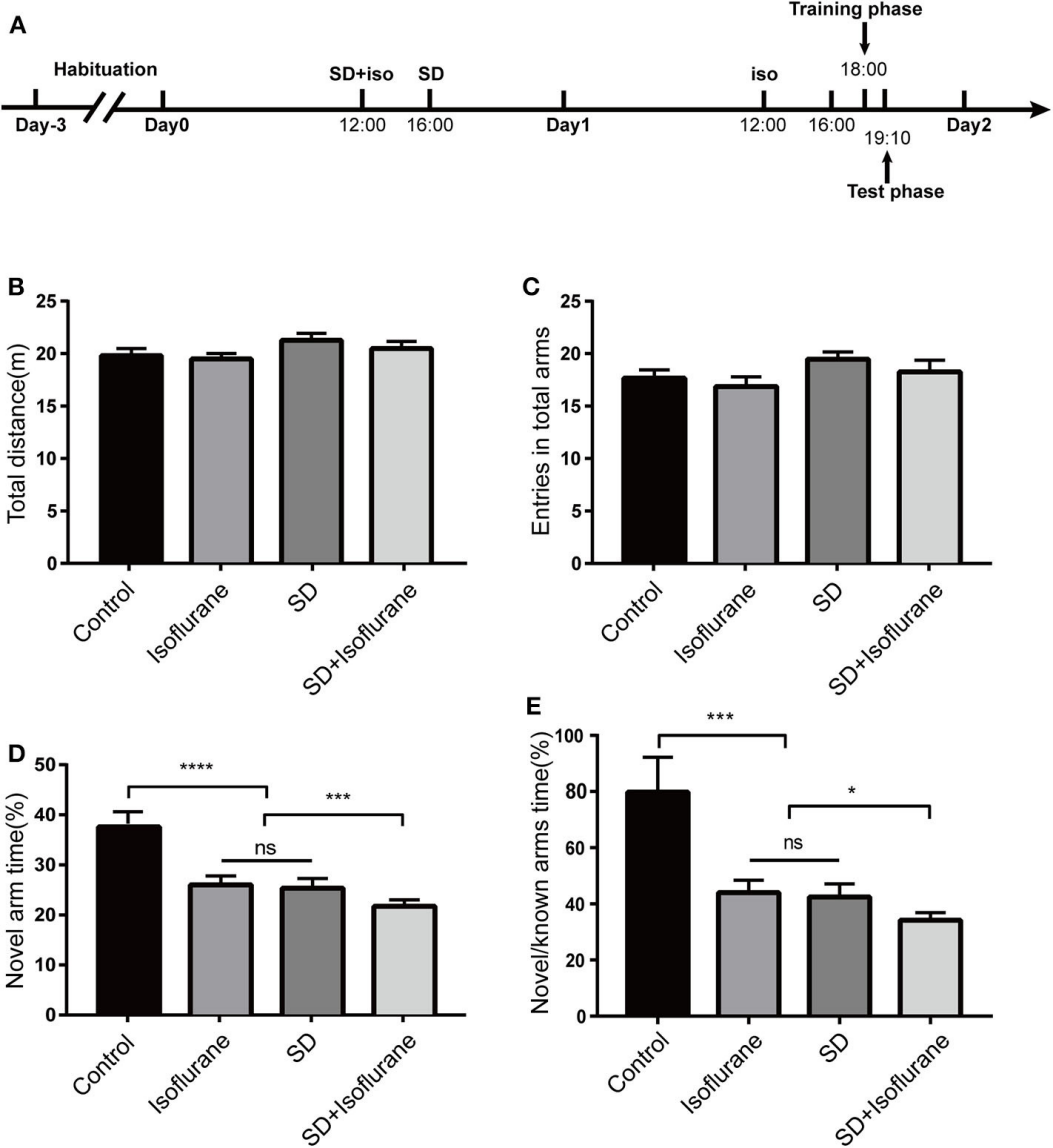
The Y-maze was used to assess short-term memory. Schematic showing the procedures for the time points that the experimental groups began to receive the treatments **(A)**. Total distance **(B)**, total arms entries **(C)**, exploration time percent in the novel arm **(D)**, and the time ratio of exploring the novel arm to the known arms **(E)**. Values are expressed as mean ± SEM. ns: no significance. *P < 0.05, ***P < 0.001, ****P < 0.0001, one-way ANOVA and Tukey test, *n* = 10/group.

Firstly, we explored whether 24-h SD would sharpen isoflurane-induced short-term memory using Y-maze test (Figure 1A). We found no difference in total distance [*F*_(3, 36)_ = 1.492; *P* > 0.05; Figure 1B], the total arm entries [*F*_(3, 36)_ = 1.532; *P* > 0.05; Figure 1C] across the four groups, which indicated that there were no significant differences in their mobility. Compared with the control group, mice in both isoflurane (*P* < 0.0001; Figure 1D) and SD (*P* < 0.0001; Figure 1D) groups exhibited an obvious lower percentage of exploration time in the novel arm, and there was no difference between isoflurane and SD groups (*P* > 0.05; Figure 1D). Mice in the SD+isoflurane group performed worse than isoflurane and SD groups (*P* < 0.001; Figure 1D). In addition, the time ratio of exploring the novel arm to the known arms in the control group was significantly higher than that in isoflurane and SD groups (*P* < 0.001; Figure 1E), and it was lower in the SD+isoflurane group (*P* < 0.05; Figure 1E) than that in both isoflurane and SD groups. These results suggested that pretreated SD followed by isoflurane exposure could aggravate the short-term memory impairment.

### Mice in SD+Isoflurane Group Showed Long-Term and Well-Trained Memory Deficits

**Figure 2:**
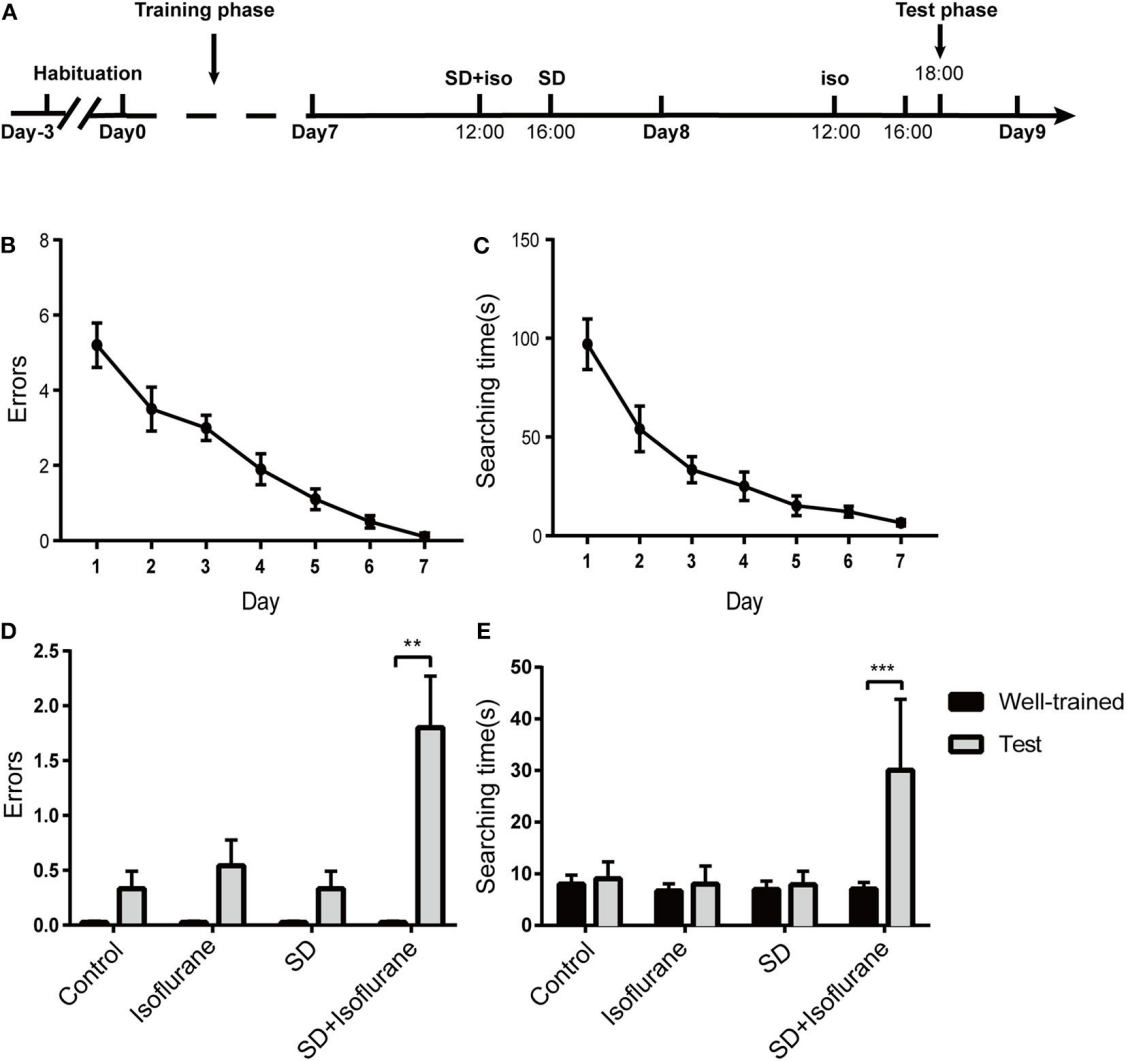
The long-term and well-trained memory were measured by the eight-arm radial maze. Schematic showing the procedures for the time points that the experimental groups began to receive the treatments **(A)**. Summary of errors **(B)** and searching time **(C)** in the 7-day training phase, RM one-way ANOVA. Summary of errors **(D)** and searching time **(E)** in the test phase. Data are presented as mean ± SEM. **P < 0.01, ***P < 0.001, paired *t*-test, *n* = 10/group.

Next, we used the eight-arm radial maze to test the retention of the long-term memory of mice in the four groups (Figure 2A). After a 7-day training, mice were well-trained to get food in the target arm within 10 s [*F*_(6, 63)_ = 163.6; 6.5 ± 0.5 s; ^****^P < 0.0001; Figure 2C] with no errors [*F*_(6, 63)_ = 21.51; ^****^P < 0.0001; Figure 2B]. There were no statistical differences in the number of errors (*P* > 0.05; Figure 2D) and searching time (*P* > 0.05; Figure 2E) after 2 h of treatments in control, isoflurane, and SD groups when compared to their own well-trained performance before the treatments. However, SD+isoflurane-exposed animals needed to explore more arms (*P* < 0.01; Figure 2D) and time (*P* < 0.001; Figure 2E) to find food. So, neither exposure to isoflurane alone nor SD alone had an effect on long-term memory, but when combined, they could impair long-term and well-trained memory.

### Sleep Deprivation Followed by Isoflurane Exposure Decreases Ca^2+^ Signals in Dorsal Hippocampal CA1 Neurons of Freely Moving Mice

**Figure 3:**
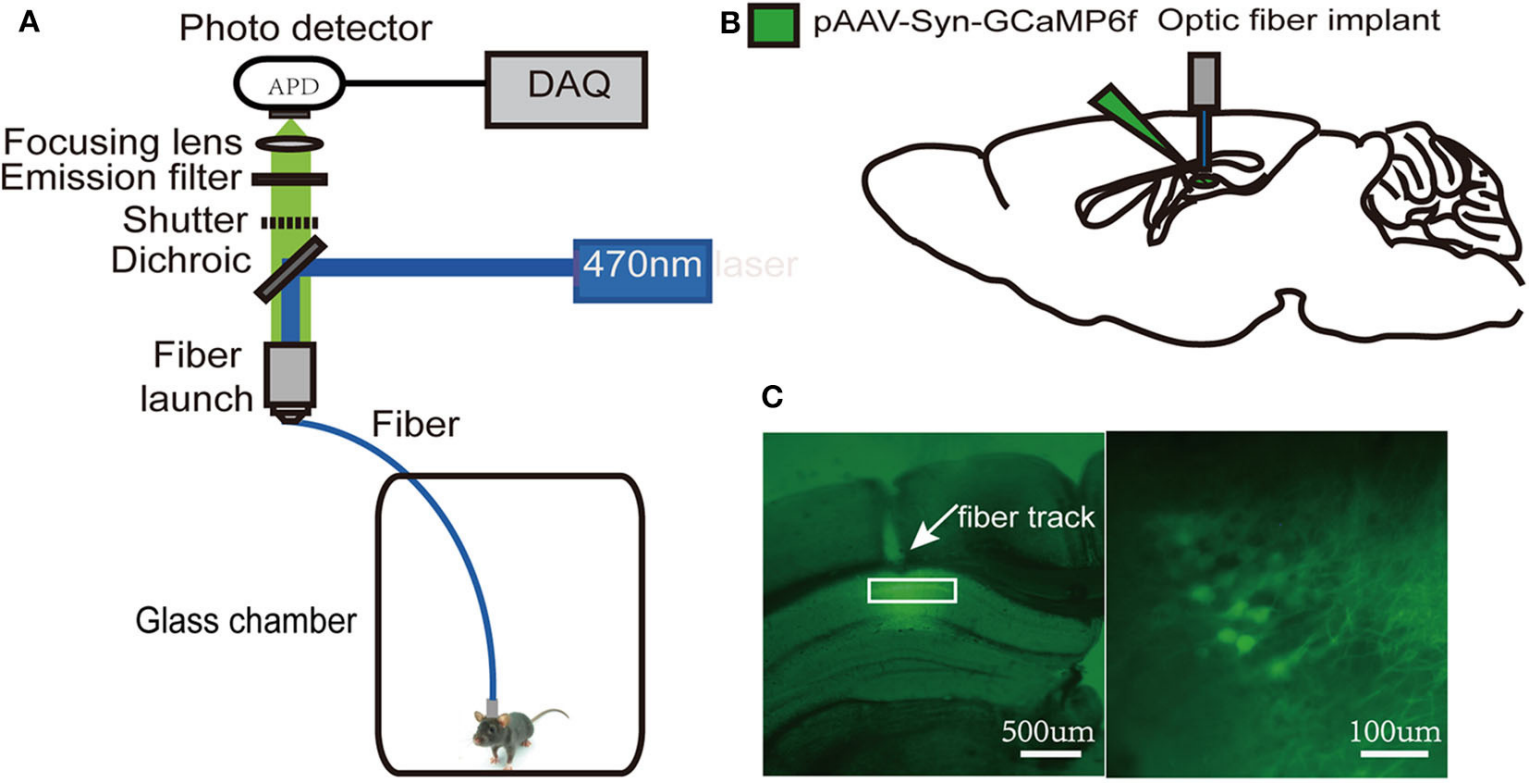
Schematic of fiber photometry Ca^2+^ recording experiment. **(A)** A custom-made fiber recording setup. **(B)** Diagram of virus injection and fiber implantation. **(C)** Histology and fluorescence images of GCaMP6f expression in dorsal hippocampal CA1 (dCA1) neurons; the white rectangle (left) denotes the area magnified at the right.

**Figure 4:**
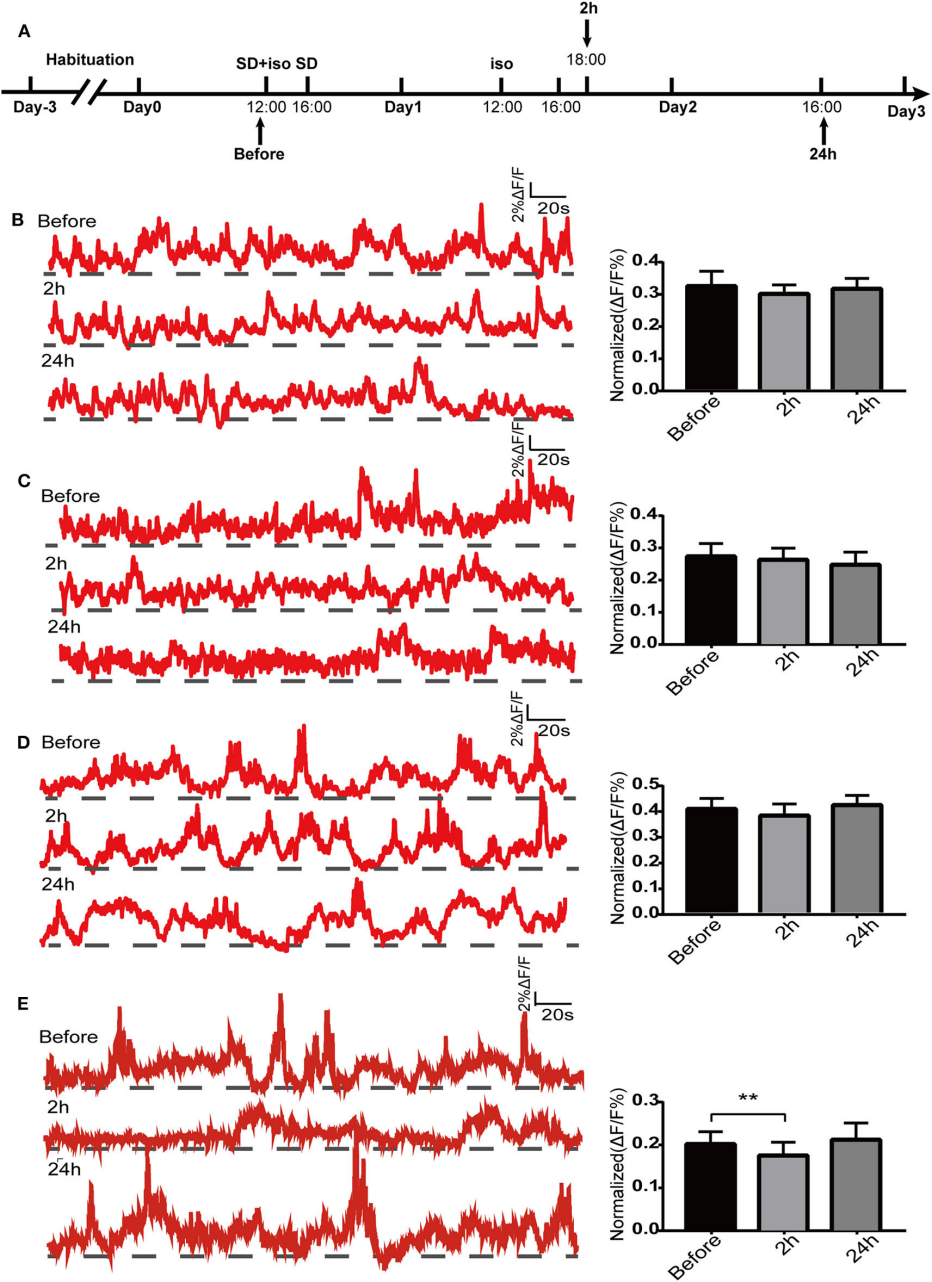
Spontaneous Ca^2+^ activity in dorsal hippocampal CA1 (dCA1) neurons of freely moving mice. Schematic showing the procedures for the time points of recordings **(A)**. The example of 5-min Ca^2+^ signal waves from one mouse (left panels) and their corresponding statistical data from seven mice (right panels) in the control group **(B)**, isoflurane group **(C)**, SD group **(D)**, and SD+isoflurane group **(E)**. Data are presented as mean ± SEM. **P < 0.01, RM one-way ANOVA and Tukey test, *n* = 7/group.

Since the dorsal hippocampus is closely related to spatial memory and cognitive function, we observed the effects of our treatments on dCA1 neuronal spontaneous activity in freely moving mice. A custom-made fiber recording device was used (Figure 3A). This involved unilateral expression of pAAV-Syn-GCaMP6f in dCA1 (Figure 3B). Neurons under the fiber track expressed GCaMP6f (Figure 3C). We recorded Ca^2+^ signals at three time points: a narrow time immediately before the treatments and 2 and 24 h after the treatments (Figure 4A). Not surprisingly, we found no significant changes in the amplitude of Ca^2+^ signals at both 2 and 24 h in the control group [*F*_(2, 18)_ = 0.1119; *p* > 0.05; Figure 4B]. In addition, neither isoflurane exposure [*F*_(2, 18)_ = 0.1168; *p* > 0.05; Figure 4C] nor SD [*F*_(2, 18)_ = 0.2502; *p* > 0.05; Figure 4D] affected the amplitude of Ca^2+^ signals. However, the amplitude of Ca^2+^ signals decreased at 2 h (*p* < 0.01; Figure 4E) in mice that suffered SD followed by isoflurane inhalation, and it was restored after 24 h (*p* > 0.05; Figure 4E).

### The Changes of Ca^2+^ Signals in Dorsal Hippocampal CA1 Neurons of Mice in the Y Maze

**Figure 5:**
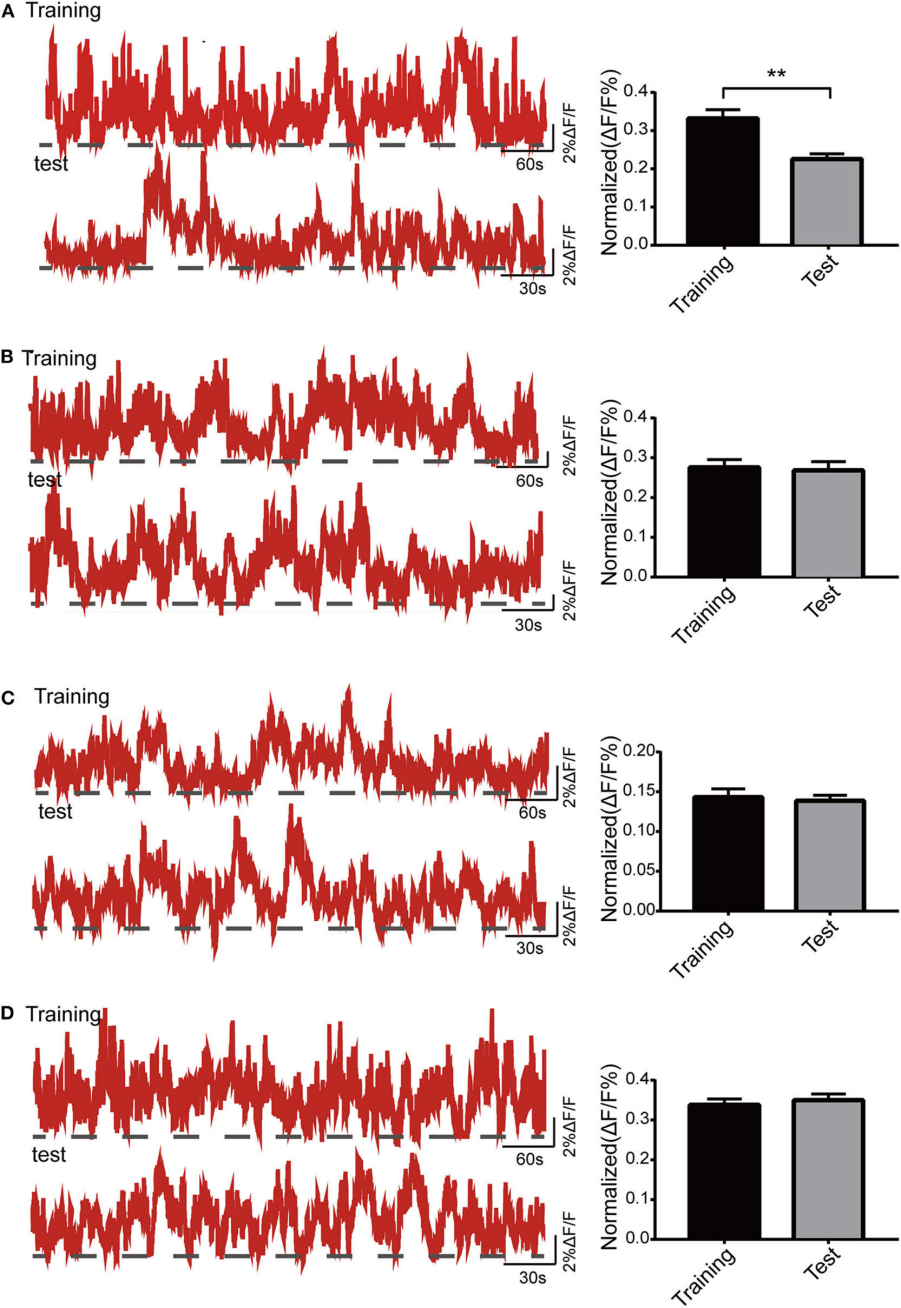
Activity of the dorsal hippocampal CA1 (dCA1) neurons in the Y maze. The example of Ca^2+^ signal waves (top: training phase; bottom: test phase) from one mouse (left panels) and their corresponding statistical data from seven mice (right panels) in the control group **(A)**, isoflurane group **(B)**, SD group **(C)**, and SD+isoflurane group **(D)**. Data are presented as mean ± SEM. **P < 0.01, paired *t*-test, *n* = 7/group.

In this part, Ca^2+^ signals were observed throughout mouse exploration in both the training and test phases. The amplitude of Ca^2+^ signals during the test period was significantly lower than that during the training period in the control group (*p* < 0.01; Figure 5A). Both isoflurane group (*p* > 0.05; Figure 5B) and SD group (*p* > 0.05; Figure 5C) showed a decreasing trend in Ca^2+^ activity during the test phase, but there were no statistical differences. The Ca^2+^ activity of mice in SD+isoflurane group (*p* > 0.05; Figure 5D) also showed no significant difference, though it was slighter higher in the test period.

### The Changes of Ca^2+^ Signals in Dorsal Hippocampal CA1 Neurons of Mice in the Radial Maze

**Figure 6:**
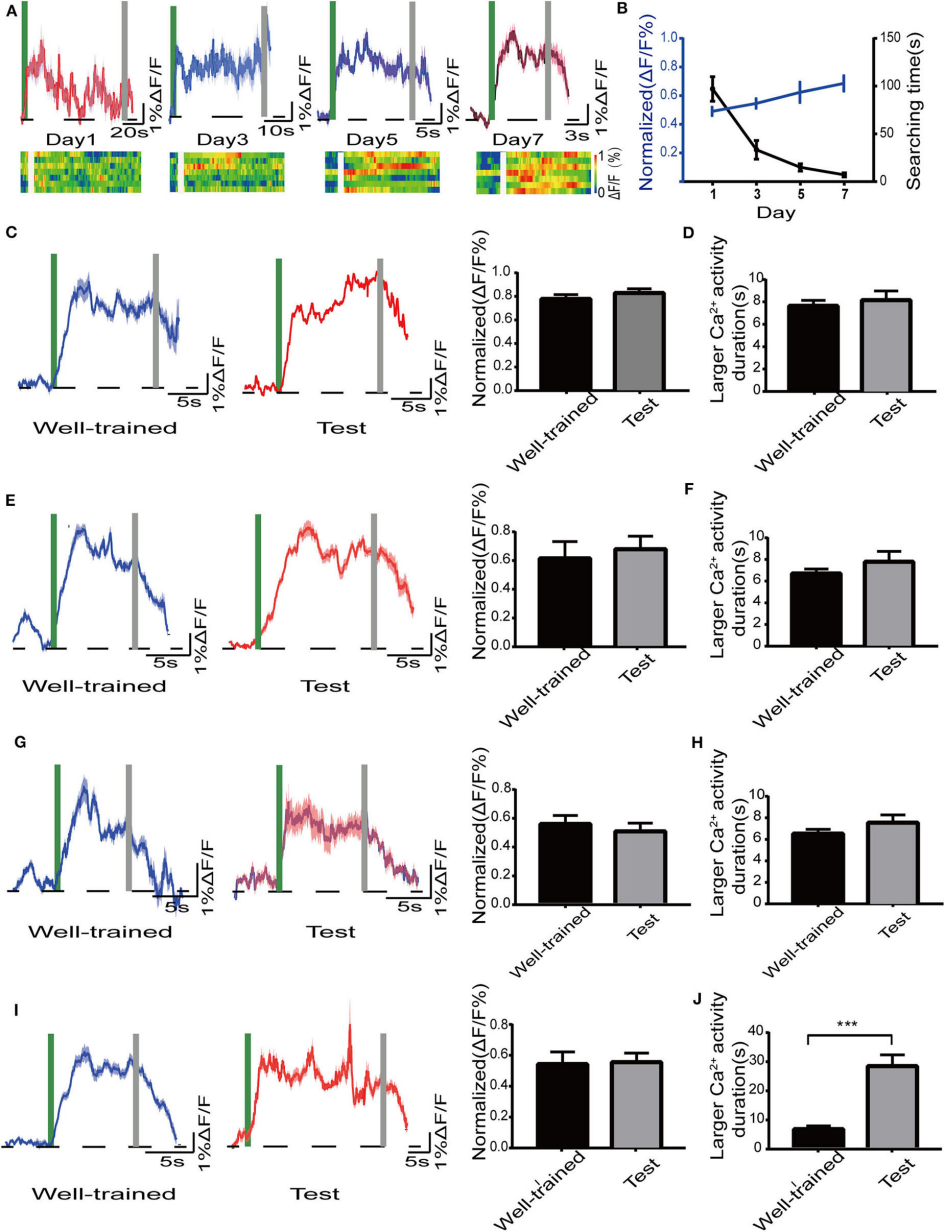
Activity of the dorsal hippocampal CA1 (dCA1) neurons in mice in the radial maze. **(A)** Mean and SEM of Ca^2+^ signals in the dCA1 neurons (top) and the corresponding heat map (lower) from seven mice during the training phase. **(B)** The plot of normalized amplitudes of Ca^2+^ signals and their corresponding searching time, RM one-way ANOVA. Mean and SEM of Ca^2+^ signals in the dCA1 neurons (left) and their corresponding statistical data (right) in the control group **(C)**, isoflurane group **(E)**, SD group **(G)**, and SD+isoflurane group **(I)** during the Well-trained and test phases. A green bar and gray bar represent the search duration. Larger Ca^2+^ activity duration in the control group **(D)**, isoflurane group **(F)**, SD group **(H)**, and SD+isoflurane group **(J)**, paired *t*-test. The duration of larger Ca^2+^ activity was defined as the period in which Ca^2+^ signals were higher than 50% of the peak ΔF/F. Data are represented as mean ± SEM. ***P < 0.001, *n* = 7/group.

We tested whether the activity of dCA1 neurons was vulnerable to our treatments in the radial maze task. Firstly, we noted that there was a mild and gradual increase in the excitability of the dCA1 neurons in the search phase as the training days progressed [*F*_(3, 24)_ = 3.005; *P* < 0.05; Figures 6A,B]. Then, mice showed a stable performance, and the dCA1 neurons produced larger Ca^2+^ signals during navigation after the end of the training days. In addition, we found a slight increase of Ca^2+^ signals when mice just found the target object in the early training days. A similar specific activity of hippocampal neurons was reported in a previous electrophysiological experiment (Fyhn et al., 2002), which might be related to encode new information. Next, we found that the activity status of dCA1 neurons remained unchanged (control: *P* > 0.05; isoflurane: *P* > 0.05; SD: *P* > 0.05; SD+isoflurane: *P* > 0.05; Figures 6C–F) during navigation between the well-trained and the test phases across the four groups, which were not in line with their behavioral performance. Finally, the duration of sustained larger Ca^2+^ activity was insignificantly different in the control group (*p* > 0.05; Figure 6G), isoflurane group (*p* > 0.05; Figure 6H), and SD group (*p* > 0.05; Figure 6I) between the well-trained and the test phases. By contrast, in the SD+isoflurane group, the duration of larger Ca^2+^ activity was much longer during the test phases than that during the well-trained phases (*P* < 0.001; Figure 6J). Together, these results showed that once the pattern of dCA1 neuronal activity formed during the learned navigation, it was hardly to be susceptible.

### Effects of Isoflurane Exposure and Sleep Deprivation on Dendritic Spines in Dorsal Hippocampal CA1 Pyramidal Neurons

**Figure 7:**
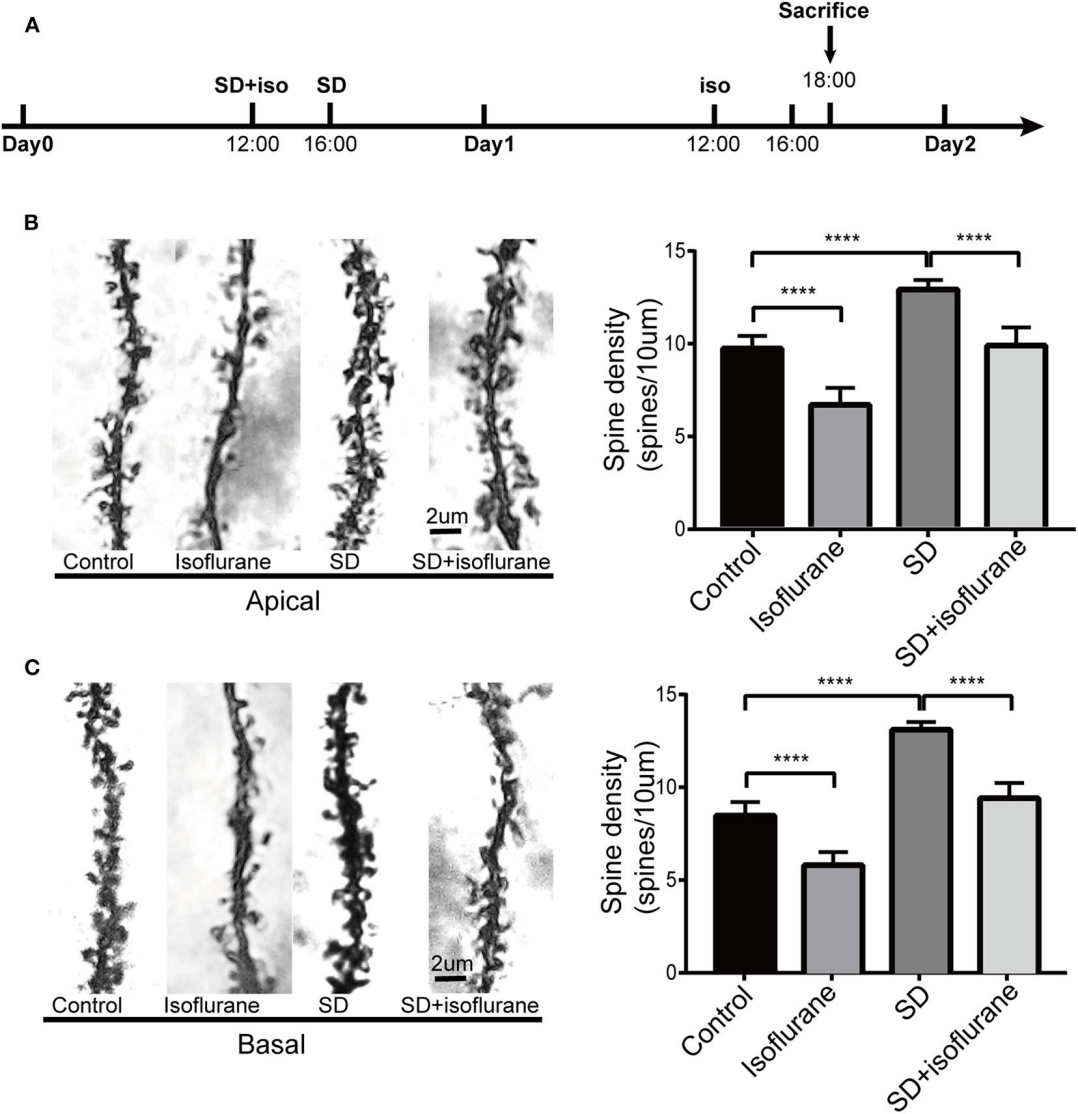
The effects of four different treatments on spine density in the second branch of dendrites of CA1 neurons. Schematic showing the procedures for the time points that experimental groups began to receive the treatments and sacrifice mice. **(A)** Changes in the apical **(B)** and basal **(C)** dendritic spine density. Representative images for spines are shown in the left panels, scale bar, 2 um. Statistical data are shown in the right panels. The results are represented as mean ± SEM. ****P < 0.0001, Tukey test, *n* = 4/group.

We observed the effects of our treatments on spine density of the dCA1 region of the hippocampus (Figure 7A). After 2 h of treatments, for the apical dendritic spines, we found that the number of dendritic spines increased (*P* < 0.0001; Figure 7B) in SD group while isoflurane lowered spine density (*P* < 0.0001; Figure 7B) compared with control mice. Spine density in SD+isoflurane group was obviously lower than that in the SD group (*P* < 0.0001; Figure 7B). For the basal dendritic spines, compared with that in the control group, isoflurane exposure significantly decreased the basal spine density (*P* < 0.0001; Figure 7C), while SD upregulated the number of spines (*P* < 0.0001; Figure 7C). The SD+isoflurane group showed a significant decline in spine density (*P* < 0.0001; Figure 7C) compared with that in the SD group.

## Discussion

This study aimed to observe the effects of pretreated SD on cognitive function and the activity of dCA1 neurons in adult isoflurane-exposed mice. Our findings indicated that lack of sleep prior to inhaling isoflurane was indeed sufficient to aggravate short-term memory deficits and induce severe damage of long-term and well-trained memory. Both treatments of isoflurane and SD failed to disrupt Ca^2+^ signals in dCA1 neurons, while SD followed by isoflurane transiently decreased the amplitude of Ca^2+^ signals when mice were freely moving. Furthermore, the density of dendritic spine in isoflurane-exposed mice with pretreated SD was lower than that in mice with treatment of SD alone. These results reveal that SD could deteriorate cognitive function in isoflurane-exposed mice by interfering with dCA1 neuronal activity and plasticity of dendritic spines.

Available evidence have shown that both isoflurane exposure (Lin and Zuo, 2011; Liu et al., 2020) and SD (Howard and Hunter, 2019; Rajizadeh et al., 2020) can impair subsequent learning and short-time memory in adult rodents. Using the Y maze, we found that both isoflurane exposure and 24-h SD affected short-term memory in adult animals, which were in agreement with previous observations, and there was no significant difference between the two treatments. Importantly, our results revealed that the short-term memory in mice exposed to isoflurane became worse once they suffered from SD. When it comes to long-term memory, there are inconsistent data on the association between it and isoflurane or SD. For the effect of isoflurane, Zhang et al. (2018) concluded that long-term memory of isoflurane-treated adult mice was not affected in different behavioral tests (Ding et al., 2016; Zhang et al., 2018). In contrast, Yang et al. (2017) argued that adult mice trained for 1 h after isoflurane performed poorly 2 days after the training phase in the fear-conditioning chamber. For the effect of SD, a recent study suggested that lemurs suffering from SD lost their capacity of long-term memory consolidation (Samson et al., 2019). It is also reported that 72-h pre-training SD impaired long-term memory significantly, while either 24-h pre-training or post-training SD had no obvious effects on long-term memory in adult rats (Nabaee et al., 2018). In the present study, we found that neither isoflurane nor SD caused a decline in long-term and well-trained memory. The possible explanation is that in our experiment, mice have been fully trained to form more consolidated memory, which may lead to gene transcription, new protein production, and growth of new synaptic connections (Kandel, 2009), and therefore, it was harder to be forgotten. The present results combined with previous findings show that the effect of isoflurane or SD on long-term memory may vary for duration of SD or anesthetic exposure, species of animal, and especially the experimental protocol of ethology tests. Most importantly, we observed that 24-h SD followed by isoflurane exposure significantly impacted risk of well-trained and long-term memory. Taken together, these results strongly highlight the importance of adequate sleep before anesthesia for the protection of later cognitive function, especially for long-term memory as it is very important for an individual's life. However, further work needs to explore how long this undesirable cognitive impairment lasts.

Given the crucial role of the dorsal hippocampus in cognitive function, we preferred to observe the effects of our processing factors on Ca^2+^ signals of dCA1 neurons in freely moving mice. We failed to find that the spontaneous activity of dCA1 neurons was affected 2 h after both isoflurane exposure and 24-h SD *in vivo*, while it decreased when the two treatments superimposed, and the change disappeared after 24 h. In addition, Ca^2+^ signals were also observed during behavior experiments. Firstly, in the Y maze, we noticed that the amplitude of Ca^2+^ signals decreased in the test phase compared with that in the training phase in the control group, which was not found in the other three groups. Since the dorsal hippocampus is primarily responsible for encoding environmental information and there are about 30% CA1 pyramidal neurons as place cells (Meshulam et al., 2017), so the possible explanation was that the better learning and memory abilities of control group mice meant that their dorsal hippocampal neurons did not have to encode environmental cues and were less active during the test phase. As for why the changes of Ca^2+^ signals in the other three groups were inconsistent with their performance in the Y maze task, the reason may be that the technique monitored the population Ca^2+^ signals and therefore the spatial resolution is relatively low that small differences in dCA1 neuronal activity between the three groups could not be detected easily. Secondly, we found that the amplitude of Ca^2+^ signals increased slowly during the training period in the radial arm maze. Previous evidence demonstrated the entorhinal–hippocampal system has a critical role in spatial memory and navigation (Buzsáki and Moser, 2013). There are two main pathways between the medial entorhinal cortex (MEC) and hippocampus, a trisynaptic pathway from the MEC layer II to dentate gyrus to CA3 to CA1 that is vital to spatial learning and memory (Zhang et al., 2013) and a monosynaptic pathway from the MEC layer III to the CA1 that is more associated with temporal association memory (Hadera et al., 2015). Qin et al. (2018) observed that the trisynaptic pathway activity changed dramatically while the monosynaptic pathway remained unchanged in the training phases in the same radial arm task. Therefore, the moderate increases of Ca^2+^ signals in the current research might result from the joint work of the two pathways. However, during the test phase, the increased Ca^2+^ signals did not decline, though the behavioral results were terrible in mice treated with SD followed by isoflurane. Electrophysiological recordings suggested that place learning could lead to an increase in the number of intermittent firing cells and the *de novo* induction of sustained firing cells during navigation (Qin et al., 2018), which could account for this sustained Ca^2+^ activity. Another reason may be that once an individual's long-term and solid memories are formed, they are transmitted to the cerebral cortex for storage, not the hippocampus (Kitamura et al., 2017). Therefore, further work needs to observe the change of neocortex neuronal activity in long-term memory deficits task.

Dendritic spines are vital to excitatory transmission and largely responsible for hippocampal synaptic plasticity (Rochefort and Konnerth, 2012). A single 20-min exposure of mature hippocampal neurons to isoflurane reversibly reduced dendritic spine density and shrank the remaining spines in rats (Platholi et al., 2014). We also found that 4-h isoflurane exposure decreased spine number. Sleep serves to decline the total number of synaptic strength after it rises during wakefulness due to external stimulus (de Vivo et al., 2017). However, Havekes et al. (2016) found that mice forced to keep awake for 5 h showed reversible decreases in spine density in the CA1 region. We found that 24-h SD increased both apical and basal spine density. Based on the results of short-term memory impairment in the SD group and a recent report that thin spines are mainly downscaled during sleep (Gisabella et al., 2020), it is tempting to believe that the increased spines in the SD group are mainly immature spines. The explanation for discrepancies with findings of Havekes et al. (2016) may lie on the duration of SD exposure or the spine density changes dynamically in CA1 region during sleep disruption, which will require further study. The result of dendritic spines in the SD+isoflurane group was caused by SD increasing immature spines and isoflurane decreasing mature spines, which was also consistent with its behavioral performance.

Overall, this is the first time to demonstrate that suffering from SD prior to isoflurane exposure could exacerbate cognitive impairment, especially long-term memory in adult mice, which is related to interference with the spontaneous activity of dCA1 neurons *in vivo* and hippocampal neuronal synaptic plasticity. Our work strongly provides references for preoperative preparation in patients under isoflurane anesthesia.

## Data Availability Statement

The original contributions presented in the study are included in the article/supplementary materials, further inquiries can be directed to the corresponding author/s.

## Ethics Statement

The animal study was reviewed and approved by the Animal Care and Use Committee of Tianjin Medical University.

## Author Contributions

Conceptualization: KZ, RD, YY, and HS. Methodology: KZ, NL, and RD. Software: KZ, CG, XD, and YL. Validation: KZ, NL, and CG. Formal analysis: KZ, XD, and YL. Investigation: SW, RD, and QJ. Resources, data curation, project administration, and funding acquisition: YY and HS. All authors contributed to the article and approved the submitted version.

## Conflict of Interest

The authors declare that the research was conducted in the absence of any commercial or financial relationships that could be construed as a potential conflict of interest.
